# Building the evidence base for urgent action: HIV epidemiology and innovative programming for men who have sex with men in sub-Saharan Africa

**DOI:** 10.7448/IAS.16.4.18903

**Published:** 2013-12-02

**Authors:** R Cameron Wolf, Alison Surdo Cheng, Laurent Kapesa, Delivette Castor

**Affiliations:** 1 United States Agency for International Development, Office of HIV/AIDS, Washington, DC, USA; 2 United States Agency for International Development, West Africa, Accra, Ghana

**Keywords:** men who have sex with men, Sub-Saharan Africa, epidemiology, HIV programmes, stigma and discrimination

## Abstract

While still an understudied area, there is a growing body of studies highlighting epidemiologic data on men who have sex with men (MSM) in sub-Saharan Africa (SSA) which challenge the attitudes of complacency and irrelevancy among donors and country governments that are uncomfortable in addressing key populations (KPs). While some of the past inaction may be explained by ignorance, new data document highly elevated and sustained HIV prevalence that is seemingly isolated from recent overall declines in prevalence. The articles in this series highlight new studies which focus on the stark epidemiologic burden in countries from concentrated, mixed and generalized epidemic settings. The issue includes research from West, Central, East and Southern Africa and explores the pervasive impact of stigma and discrimination as critical barriers to confronting the HIV epidemic among MSM and the intersecting stigma and marginalization found between living with HIV and sexual minority status. Interventions to remove barriers to service access, including those aimed at training providers and mobilizing communities even within stigmatized peri-urban settings, are featured in this issue, which further demonstrates the immediate need for comprehensive action to address HIV among MSM in all countries in the region, regardless of epidemic classification.

The HIV epidemic in sub-Saharan Africa (SSA) is dynamic with regional and temporal variation. The 2012 Report on the Global AIDS Epidemic reports a decline in HIV incidence among the general population (GP) by 25%, a decrease by 40% in HIV-related mortality, and that more than half of people living with HIV (PLHIV) who are eligible for treatment were on treatment in SSA [1]. The UN classifies countries as *low level* when HIV prevalence in the GP, as measured by HIV surveillance data, is under 1% and key population (KP) prevalence does not exceed 5%, where KPs are defined as female sex workers (FSWs), men who have sex with men (MSM) and people who inject drugs (PWID); *concentrated* epidemics are where HIV prevalence is under 1% in the GP, but any KP (e.g., FSW) prevalence consistently exceeds 5%; and *generalized* epidemics are where HIV prevalence exceeds 1% in the GP regardless of HIV status among KPs [1,2].

An epidemic appraisal proposed by Wilson and Halperin and others that characterizes the typology of HIV transmission within the country, rather than simply crude estimates of incidence and prevalence at the national level, called for reclassification of countries based on transmission dynamics [3,4]. They posited that *concentrated* epidemics are driven by KPs, and they added *mixed* epidemic settings, where both the GP and KPs play a role in HIV transmission, and *generalized* epidemics, in which they argued that contribution to new infections from KPs is insignificant. But this is debatable.

UNAIDS estimates HIV prevalence to be 17.9% among MSM in SSA. Yet there are limited epidemiological data for MSM or KPs in general in this region. For programmes to be well aligned, we need to better understand the epidemiologic and behavioural burden and social drivers of HIV within KP groups in all epidemic settings. The articles in this series focus on building the literature on epidemiology, social drivers of transmission and programmatic innovations for MSM in four regions of SSA: West, Central, East and Southern Africa. Respondent-driven sampling (RDS) methods were used to engage networks of MSM in these pioneering studies, which characterize the HIV epidemiology among MSM in Cameroon, Senegal, Malawi and Swaziland as elevated and sustained when compared to men in the GP. This supplement also includes a meta-analysis of prevalence studies from KPs in Central and West Africa, which helps to ground our understanding of MSM within the broader context of KPs.

The extraordinary burden of internalized and external stigma and discrimination creates the paralyzing barriers to MSM access for prevention, care and treatment services that are notable across these studies. Key dynamics of intersecting stigmas of HIV and sexual orientation among HIV-positive MSM are also explored in Swaziland [5]. Structural interventions to address the pervasive stigma in healthcare settings have been called for by the public health community, and this supplement also addresses this issue with findings from sensitivity trainings in coastal Kenya, which can influence and support clinical work with MSM who come to healthcare settings for sexually transmitted infection (STI) and HIV-related care and treatment. Additionally, innovative strategies for community mobilization engaging peer leaders in small-group safe spaces within stigmatized peri-urban townships are addressed through the article from South Africa.

While the role of sex among men is increasingly described in concentrated epidemic settings, studies from Southern Africa within *generalized* epidemics, where KPs are conventionally not thought to play a significant role (e.g., South Africa, Swaziland, Lesotho, Malawi, Namibia, Botswana and Zimbabwe), have also shown MSM to have high prevalence of HIV, syphilis and hepatitis B virus, with disease burdens equal to or greater than those of men in the GP. Still, because of the conception of KPs as insignificant in these generalized epidemic settings, any data collection or targeted response is limited [6].

Swaziland has been documented to have the highest HIV prevalence globally. The incidence of HIV appears to have peaked in 1998–1999 at 4.6% according to UNAIDS estimates, while in 2009 it was estimated to be 2.7%. Recent data from the Swaziland HIV Incidence Measurement Survey (SHIMS) estimated HIV incidence at 2.4% in the total population: 3.1% among women and 1.7% among men [1,7,8]. The 2009 Swaziland Modes of Transmission (MOT) study characterized major drivers of incident HIV infections to be multiple concurrent partnerships before and during marriage as well as low levels of male circumcision [9]. While these drivers were validated through the SHIMS, it is critical to note that like many MOT studies in generalized settings in Africa, there was no known prevalence among FSWs or MSM, so the MOT analysis assumed a low frequency of both practices and therefore assigned them as minor drivers of the epidemic.

In this issue, Baral and colleagues conducted the first cross-sectional study to estimate HIV prevalence and its risk factors among MSM in Swaziland [10]. The HIV prevalence in the RDS sample of 324 MSM was 17.6%, and the odds of HIV prevalence increased by 20% for each year of age. The vast majority (70%) of the sample reported being unaware of their HIV status, and consistent condom use with lubricants with male partners was reported by 12.6% of respondents. The authors note that within their MSM sample, HIV prevalence is consistent with that of an age-matched sample from the GP until age 24–26 years, when the prevalence of HIV among MSM rises *higher* than that of other men in the GP – with HIV prevalence of 43.1% among MSM older than 27 years [10].

These data as well as other recent data showing alarming rates (70.3%) among FSWs have called researchers to rethink the prevention, care and treatment response in Swaziland [11]. Similarly, in Malawi, a high-HIV-burden country in East Africa, Wirtz *et al*. conducted the most comprehensive MSM study to date in Malawi, where the HIV response has largely focussed exclusively on heterosexual and vertical transmission of HIV and where an estimated 8% of GP men have HIV [12,13]. A sample of 338 Malawi MSM had prevalence of HIV and active syphilis of 12.5 and 4.4%, respectively, after adjusting for RDS approaches. Ninety percent of HIV infections were previously undiagnosed, and about half reported consistent condom use with casual male partners. Among MSM 26 years and older, prevalence of HIV was 28.1%.

West and Central Africa, the most populous regions of Africa, have a mixture of HIV epidemics, and KPs are better understood to play an important role in the overall transmission dynamics in Nigeria, Senegal and Burkina Faso, where KPs consistently show elevated HIV prevalence in comparison to the GP, as reported by Papworth *et al*. in a meta-analysis of KPs from West and Central Africa [14]. In Cameroon, where the GP HIV prevalence for men is 2.9%, Park and colleagues sampled 511 MSM in Douala and Yaoundé through RDS and estimated the HIV prevalence to be 37% [15]. In Douala and Yaoundé, respectively, HIV prevalence was 25.5 and 44.4% [16]. Like in the other studies, the sample was young, with a median age of 24 years, and as age increased, HIV rates increased at staggering levels. HIV among MSM aged 24–29 years was 47%, and it was 49.4% for those over 30. About half of respondents did not use condoms consistently with casual partners (48.5%), and even more did not use them consistently with regular partners (64.1%). Similarly striking in Senegal, where GP prevalence among reproductive age men is 0.5%, the article included by Drame *et al*. reports baseline HIV prevalence among a sample of MSM at 36.0% (43/114) with cumulative HIV prevalence after 15 months at 47.2% (51/108) [17].

Due to the criminalized nature of male-to-male sex in all countries where studies from this issue took place, with the notable exception of South Africa (which still experiences high stigma), MSM are often afraid to visit healthcare services; and when they do go, they are reluctant to disclose their sexual histories to healthcare providers for fear of rejection, derision, or other negative reactions. Continued work is needed to develop violence screening, reporting and mitigation approaches within these settings [18]. The authors in this issue demonstrate that access to and coverage of quality HIV services for MSM are still marginal and not sufficient to reverse the epidemic's trend among MSM, and this is aggravated by several factors, primarily stigma, discrimination and limited domestic investment in programmes focussed on MSM [19–21]. Lessons learned from mature programmes targeting FSWs in the region show that with limited coverage, poor dosage and an inadequate combination of approaches, even proven interventions will be ineffective [22].

Research focussing on MSM sexual risk behaviour in SSA is scant, and even less is known about the stigmatization and discrimination of HIV-positive MSM [23]. Kennedy *et al*. conducted 40 in-depth interviews from 20 HIV-positive MSM, 16 interviews with key informants and three focus group discussions with MSM community members. Internalized and experienced stigma was high among the men living with HIV, who report that they conceal their HIV status from others. MSM living with HIV reported experiencing greater social isolation and lack of support for care-seeking and medication adherence. Perceived and experienced stigma from healthcare settings led to delayed care seeking and travel to more distant clinics to retain anonymity at home. The authors argue that mental health interventions, training for healthcare providers and better protection against discrimination are needed for Swazi MSM living with HIV, which corroborates previous findings from South Africa [24].

The association between HIV prevalence and the existence of community-based HIV interventions targeting MSM and other KPs has been well described [25]. Tailored community-based programmes that provide MSM with high-quality, sensitive services that are socially and economically acceptable and led by the beneficiaries themselves is a promising approach. The current article by Batist *et al*. expands on this association with the use of safe spaces to remove barriers to service access, including those aimed at training providers and mobilizing communities even within stigmatized peri-urban settings, which led to greater feelings of connection, social support and self-esteem among MSM community members and also led to these spaces becoming distribution points for condoms, lubricants and HIV education.

Also in this supplement, two articles look at a sensitivity training for healthcare workers providing services to MSM [21,26], highlighting the pre-existing attitudes that can manifest during clinical encounters with MSM. Healthcare workers in SSA generally do not receive specific training in working with MSM or other KPs, and they may not be aware of risk factors for HIV transmission or appropriate care and treatment needs. Healthcare worker training has been identified as a priority intervention to support a minimum package of essential services for MSM [27]. In van der Elst *et al*., the researchers implemented a novel approach to sensitivity training for healthcare workers providing services to MSM [21]. The training consisted of self-directed, publically available online modules followed by group discussions focussed on MSM sexual risks and healthcare needs. Knowledge and homophobia were assessed prior to training, immediately after training and three months post training. There was a statistically significant decline in homophobia sustained after three months post training, with greater reductions for males and those in clinical roles (doctors and nurses), who were also more likely to have higher homophobia scores pre-training. However, it remains to be seen whether these attitudes can be maintained over time without ongoing support [21].

In a subsequent article in this supplement, van der Elst *et al*. explored topics including the sexual identification of subcategories of MSM, sexual practices and risks for HIV and STI transmission, practices for sexual history taking and sexual health examinations for MSM [26]. Stigma was also a concern for healthcare workers, such as negative judgements from peers or community members for being associated with MSM, and was an ongoing challenge after the training. After completing the programme, healthcare workers expressed greater acknowledgement of MSM patients in their clinics, empowerment to address their needs, and a better understanding of the biological, behavioural and social influences that lead to HIV and STI risk for MSM.

The term “MSM” is meant to address all MSM, regardless of their gender or sexual identities. Some MSM self-identify as heterosexual rather than gay, homosexual, or bisexual, especially if they also have sex with women, are married, only take the penetrative role in anal sex, or have sex with men for money or convenience [28]. They may not consider their sexual encounters with other men in terms of gender identity or sexual orientation, or they may more aptly self-identify using local social terms which reference sexual identities, masculinity and femininity, and behaviours. One noteworthy finding within the articles presented in this issue from Malawi and Swaziland was the disconnection between gender identity and sexual orientation [10,12,20]. While nearly all the respondents of both surveys reported that they were either gay or bisexual and had anal sex with men as criteria for eligibility in the study, a sizeable number reported that they were not male. In Malawi, 17.0% reported they were female, and another 2.8% said they were transgender. In Swaziland, 15.7% reported being female, and 1.8% said they were both male and female. It is not clear whether participants actually considered themselves to be women or whether their sexual behaviour caused them to consider themselves not to be men. There is a need for further study to better understand how these terminologies translate into risk and sexual identity profiles while not singling out these individuals for further stigma.

As more data become available for MSM in SSA, including in Southern African generalized settings, MSM needs should be identified and addressed throughout the continuum of HIV prevention, care and treatment. We continue to need evidence-based interventions to identify, create and train healthcare providers as well as community champions, including lawyers, media owners, journalists and religious leaders sensitive to MSM programming, and establish community-driven programmes while expanding integration within the health system as appropriate.

The articles in this series have shown that throughout SSA, there is a significant and sustained epidemic among MSM, fear of discrimination from healthcare settings, and provider-based and self-stigma which impede prevention, care and treatment [29–31]. The findings highlight the need to focus on MSM as a critical KP in “mainstream” approaches as well as MSM-targeted models. Countries in Africa characterized as having generalized epidemics, where KPs are not considered relevant, must be re-conceptualized based on these findings. All “generalized” epidemics are in reality *mixed* epidemics with ongoing transmission among KPs, and this becomes increasingly clear as GP prevalence rates decline while MSM experience expanding epidemics.

While it is not clear what proportion of new HIV infections are linked to MSM directly and via second-order transmission among their partners, it is clear that without addressing this underserved, stigmatized population, HIV transmission will be impossible to abate. Therefore, the benefits of targeted structural, behavioural and biomedical services for MSM go beyond the individuals to benefit the larger public welfare and security of all countries within SSA as well as globally. After three decades of the fight against HIV, plans to end the HIV epidemic through goals such as the AIDS-free generation and the US President's Emergency Plan for AIDS Relief's (PEPFAR) Blueprint seem possible. Substantial progress has been made, and more will come through vigilance, courage, tolerance and commitment.
